# Development, implementation and evaluation a palliative care program for colorectal cancer patients: a mixed methods protocol study

**DOI:** 10.1186/s12885-022-09538-9

**Published:** 2022-04-22

**Authors:** Masoud Bahrami, Masoumeh Masoumy, Alireza Sadeghi, Rohallah Mosavizadeh

**Affiliations:** 1 Cancer Prevention Research Center, Department of Adult Health Nursing, Faculty Of Nursing and Midwifery, Isfahan University of Medical Sciences, Isfahan, Iran; 2 Student Research Committee, Faculty Of Nursing and Midwifery, Isfahan University of Medical Sciences, Isfahan, Iran; 3 Cancer Prevention Research Center, Department of Hematology-Oncology, Faculty of Medicine, Isfahan University of Medical Sciences, Isfahan, Iran; 4 ALA Cancer Prevention and Control Center, Department of Islamic Education, Faculty of Medicine, Isfahan University of Medical Sciences, Isfahan, Iran

**Keywords:** Palliative Care, Colorectal Cancer, Mixed Methods, Protocol Study, Nursing

## Abstract

**Introduction:**

Colorectal cancer(CRC) patients are among the incurable groups who need comprehensive palliative care covering all aspects including physical, mental, social, and spiritual**.** The purpose of this study is to develop, implement, and evaluate a holistic palliative care program for CRC patients in order to improve quality of life of CRC patients.

**Methods:**

This study is an exploratory mixed methods study which will be conducted using a sequential qualitative-quantitative design (QUAL quan) consists of four sequential steps using the approach proposed by Ewles & Sminett to develop the program. In the first phase, a qualitative study (semi-structured interview) will be conducted to discover the needs of CRC patients from the perspective of patients, family members and care providers. In the second phase, the literature review will be performed with the aim of confirming and completing the discovering new needs. In the third phase, in order to prioritize the identified needs and prepare a initial draft of the palliative care program will be done a panel of experts. In the fourth phase, the part of the developed program according to the opinions of the panel of experts, will be implemented as quasi-experimental intervention and the effect of intervention on quality of life will be evaluated.

**Discussion:**

This results of this study are expected to meet the needs of CRC patients and their families through providing a holistic care and improve their quality of life in the socio-cultural context of Iran. This program can be useful in providing care, education, policy making and for future research.

## Background

CRC is the third most common cancer and the second deadliest cancer worldwide [1]. In Iran, CRC is the third most common cancer among men and the second most common cancer among women [2]. There is a significant increase in the prevalence of CRC in Isfahan from 2011 to 2015. Based on these statistics, CRC is an serious health problem in the country, especially in Isfahan province [3]. Knowing about one's cancer is a shocking and worrying experience which can lead to various physical, psychological, social, financial and spiritual problems. These patients experience physical treatment-caused complications and psychological problems including anxiety, fear, depression and mental imbalance. Moreover, multiple hospitalizations for treatment can prevent the continuation of normal life and return to work, and lead to the feeling of isolation and loss of independence in these patients [4–6]. Patients' psychological problems lead to reduced quality of life, lack of adherence to treatment and poor prognosis [7]

Additionally, the population CRC survivors is increasing day by day [8] This means that improving the survival of patients imposes new challenges such as caring for the long-term complications of the treatment and maintaining the quality of life of patients upon the care provider [9].Therefore, unlike the past, today the quality of life is assessed as one of the important outcomes and indicators determining the effect of treatment in patients. Improving the quality of life of cancer patients has been one of the main goals of care and treatment of cancer patients [10].

Therefore, the troublesome symptoms and complications of cancer and its treatments, on the other, and the huge treatment costs imposed on the patients and their families, make these patients in need of quality-enhancing care. Accordingly, these patients are among the incurable groups who need holistic care in the form of "supportive and palliative care" [11]. In fact, palliative care includes all active and complete measures which are taken to reduce pain and improve the quality of life of patients [12]. According to studies, patients at different stages of CRC need palliative care to improve their symptoms, as 30% of CRC patients at the time of diagnosis are in the advanced stages of the disease and 40% to 65% of all patients with CRC are in the metastatic stage [13]. Studies have confirmed the benefits of integrating palliative care into oncology. What the results of these studies show are an improvement in patients' quality of life, less psychological distress, less aggressive end-of-life care, and more patients and caregiver satisfaction with the received care [14] In other words, providing palliative care in the form of specialized services may lead to better control of the symptoms, pain management, reduced patient and family anxiety and provision of high-quality care, the reduction of unwanted hospitalizations, improved quality of life and reduced economic burden of cancer on the health system [15, 16].

Depending on which part of the intestine is involved in cancer, ostomy bags will be placed for patients and patients with ostomy will suffer from various physical, psychological, social, financial and sexual problems in the long-term. Because this ostomy bag and the annoying smell and sound of this bag are always with them, as a result, the quality of life of patients and their families will decrease despite disease control. Therefore, designing a specific palliative care program for these patients is necessary due to having different problems caused by digestive or ostomy problems.

The high prevalence of CRC in Iran is an important health problem for the country, and patients with this disease need palliative care services at all stages of the disease along with the main treatments. Therefore, it is necessary to identify the needs of CRC patients in Iran based on the country's characteristics and underlying conditions and, then, design and implement a program tailored to the needs of these patients. Therefore, in order to provide integrated and comprehensive palliative care to these patients, both methods (qualitative and quantitative) will be used.

### Objective

The aim of this study is designing, implementing and evaluating palliative care program for CRC patients.

## Materials and method

This is an exploratory sequential study(QUAL quan) will be done in 4 steps. Ewles & Sminett (2010) model will be used to develop the program [17]. Studies that explore the CRC patients' needs have not been performed in Iran, so in this study, the qualitative stage has more priority.

### Phase1 qualitative study

A qualitative study, using in-depth semi-structured interviews and field note and memoing will be conducted to discover and identify the needs of colorectal cancer patients and challenges and strategies to meet needs from the viewpoint of the patients, their families and health care providers.

### Study participants

The participants in this phase will include CRC patients, their family members and health service providers including oncologists, oncology nurse, psychologist, spiritual caregiver, social worker, and nutritionist. The purposive sampling method will be used with maximum diversity in sex, age, education level, occupation.

### Inclusion criteria

#### Colorectal cancer patients

- desire to participate in the study
- undergoing chemotherapy and radiotherapy or having received them
- being in the stages 2 and 3 of the diseases and not being in the metastasis stage and end stage

#### Family caregivers

- desire to participate in the study
- who have experienced continuous care of a patient with colorectal cancer at home

#### Health Care Providers

- have worked for at least 6 months in adult cancer or palliative care wards
- desire to participate in the study

### Exclusion criteria

- Reluctance to continue cooperation
- withdrawal from the study at any stage of the research

### Procedure

The study setting includes cancer treatment centers and MACSA in Isfahan. The data will be obtained through in-depth semi-structured interviews using interview guide questions. For ethical considerations, the purpose of the study will be explained to the participants before the interview and informed written consent will be obtained from the participants for recording the interviews, and the place and duration of the interviews will be determined according to the preferences of the participants. Guiding questions will be extracted from the relevant literature and provided in 3 parts by considering the related participants groups.

The first part will be comprised of the questions related to CRC patients to identify needs, problems and concerns and requires health services. The data obtained from this section will provide useful information about needs and challenges to meet the needs of these patients.

The second section is the questions related to family caregiver. The purpose of these questions is to identify the impact of the disease on various aspects of your patient's life, your patient's needs after diagnosis CRC. HCP questions will be in the third section. The questions in this part are to identify cares and services required by these patients when providing care to these patients. The interviews which will be continued until data saturation. Data analysis will be performed simultaneously with data collection using the qualitative content analysis based on Graneheim and Lund man approach [18].The interviews will be transcribing verbatim and the researcher will read the text of the interviews several times. Then, the main idea in important sentences and items will be tagged as code. After the same initial codes will be put together, the data will be reduced and finally categories of the codes will appear. The MAXQDA 10 software will be used for the management of the data. In order to trustworthiness of the qualitative data of four criteria including credibility, transferability, dependability and confirm ability are used [19]. The following measures include selecting participants with maximum variation, allocating sufficient time to data collection and using various data collection methods such as interviews and field observations and check the accuracy of the researcher's perception through review a sample of the codes by an outsider observer and participants will be increase credibility. By comprehensive describing all the steps of the research so that other researchers can do the same steps will be increase transferability. In order to increase confirm ability, the study steps will be accurately recorded so that a thorough audit of the work is possible.

### Phase2 literature review

In this phase, the researcher will be conducted searching multiple electronic databases including Scopus, MEDLINE, Proquest, Web of Science(SID), Embase with mesh-matching keywords like the “Palliative Care”, “Colorectal Cancer”,"need", "unmet needs" will be searched for the related articles from 2010 to 2021. Persian databases in SID, Magiran and Iranmedex will be searched with the Persian keywords for the related articles from 2010 to 2021. The purpose of this phase is to confirm the needs discovered in the qualitative phase or to discover the new needs and categories and to discover the strategies to meet the needs of CRC patients in related texts.

### Inclusion criteria

- The full text of the original articles (quantitative, qualitative and mixed methods), review articles, book reviews
- Articles published between 2010 to 2021
- Articles are published in English or Persian languages

### Exclusion criteria

- Articles related to advanced CRC patients/survivors’needs
- Abstracts, short communications, letters to the editors
- articles without having access to their full text

### Phase3 development of palliative care program

In this step, Ewles & Sminett model (2010) will be used to develop the program, which the steps of this model include identifying needs and prioritize needs, identifying general and specific goals, selecting the best strategies to achieve specific goals, identifying resources, evaluation methods of program, and determining intervention activities. In order to prioritize needs and to identify goals and selecting the best strategies and to identify evaluation methods and type intervention will be used Experts panels.

### Study participants

The member of experts will include oncology nurse, Oncologist, Physicians, Psychologists, Social workers, Nutritionists, Spiritual caregiver, faculty members and other experts who provide care to CRC patients.

### Inclusion criteria

- Having experience and expertise in care of CRC patients
- Having experience in palliative care, CRC and cancer studies
- Desiring to participate in the panel sessions.

### Exclusion criteria

- Reluctance to participate in panel meetings
- Failure to attend in at least two sessions.

### Procedure

Expert panel members will be selected using purposive and snowball sampling methods. The number of participants will be at least ten [20]. Before the meeting, a list of extracted palliative care needs and the goals of the panel session will be provided to specialists. At the beginning of the session, the researcher will provide a summary of the research methodology as well as the data obtained from the qualitative study and literature review and objectives of the panel session. Then of Experts group will be asked to score needs and strategies based on criteria of "importance", "cost", and "applicability" in 5-point Likert (1 lowest and 5 highest) [21]. Then, experts groups will discuss the needs and priority strategies as well as the type of intervention to meet the needs of these patients and the evaluation methods of the intervention and will present their suggestions. The researcher will record and note the stated topics in order to design the program. Once the priority needs and strategies have been identified, the initial draft of the palliative care program for CRC patients will be developed. Then the initial plan prepared to confirm the content will be sent via email to the same specialists participating in the panel meeting, and after receiving the experts' suggestions, the necessary corrections will be made and the developed palliative care plan will be finalized.

### Phase 4 program implementation and evaluation

In order to determine the effectiveness the part of program focuses on meeting the needs and improvement quality of life of CRC patients, a pretest–posttest quasi‑experimental design will be implemented.

### Study participants

All patients with CRC will be included. Patients will be selected using convenient sampling [22].

### Inclusion criteria:

- patients has been diagnosed in the stages 2 and 3 disease
- patients undergoing chemotherapy and radiotherapy or those who have received these treatments
- have not existence of metastasis

### Exclusion criteria

- Reluctance to collaborate at any stage of research
- Absence in 2 sessions of intervention sessions

### Procedure

After receiving the code of ethics from the ethics committee of IUMS, the researcher will go to the research environment that will be included hospitals centers providing care to CRC patients as well as palliative care centers in Isfahan. In this study, people who meet the inclusion criteria will be selected by convenient sampling method, so that the researcher written informed consent will be taken from the samples and the samples will be randomly assigned to the control or intervention groups. The number of samples will be calculated based on the formula of the sample size. Considering the probable 10% drop in the samples, 30 CRC patients will be allocated to each group.

$$n=\frac{2({z}_{1}+{z}_{2}){\left(s\right)}^{2}}{{\left(d\right)}^{2}}$$

After determining the type of intervention according to the experts group comments, in the intervention group, the intervention will be implemented, and the control group will not receive any intervention. After completing the intervention sessions for the intervention group, a similar package will be provided for the samples in the control group. Impact of intervention will be evaluated using the standard questionnaires will be completed by both groups, before and after the implementation of the intervention.

The tools will be used to collect information including demographic questionnaire and QLQ-CR29 questionnaire and also in accordance with the type of intervention performed other valid and reliable questionnaires will be used to evaluate the effectiveness of the intervention.

QLQ-CR29 questionnaire is the quality of life questionnaire for colorectal cancer. This questionnaire belongs to the EORTC [23]. It is a self-administered tool consisting of 29 questions designed to assess the quality of life of CRC patients. The answers in the form of a 4-point Likert scale range from 1 to 4, and the final score of the questionnaire for the performance indicators ranges from zero (worst health condition) to 100 (best health condition) and for the symptom indicators from zero (no symptoms) to 100 (maximum symptom). The questions of the questionnaire are related to the patient's condition in the past week, except for sexual function which is related to the last four weeks [24].The validity and reliability of QLQ-CR29 have been measured in Khazaeli study (2013) and the total Cronbach's alpha of the questionnaire has been equal to 0.89 [25].

### Data analysis method

The data will be analyzed using descriptive and inferential statistical methods in SPSS20 software.

### Integration of data

In this research, integration of data will be conducted by quantitative and qualitative results will be expressed together in the discussion.

## Discussion

Iranian society is transitioning from experiencing various changes in social aspects, including changes in the lifestyle of people, changes in the population pyramid, such as population aging, the reduction of the age of diseases, and new methods of treatments for chronic diseases which affect the survival and health status of people. To reach new horizons and deal with these challenges, the most important of which is CRC, the service delivery system must show an appropriately and timely response to these changes.

Providing palliative care is considered as one of the main necessities of the health system, especially in cases where CRC and the rate of the related deaths are high and, thus, there is a need for specialized and palliative care, pain management and symptom control. In the area of providing palliative care, the philosophy and mission of this care is the same in all parts of the world. Assessing the needs of the patients, palliative care is based on providing comprehensive and holistic care for patients. Therefore, this study is a turning point in providing comprehensive care to CRC patients based on needs, patients' experiences in socio-cultural conditions by developing a palliative care program [26].

The program developed in this study can be given to members of the health team as a guide to improve the quality of life of patients with CRC. Developed based on a holistic approach, this program will help members of the health team to provide care in the form of an inter-professional team. Providing care by using this program helps to pay more attention to the necessity of a holistic view to patients and their family through considering their various physical, psychological, spiritual and material dimensions in their care, provide the patients with better services and satisfaction, reduce psychological distress, and improve the quality of life of CRC patients. Moreover, based on the developed program, strategies and procedures for meeting the different needs of CRC patients will be extracted by reviewing the related literature and interviewing patients, their families and members of the health team. Using these strategies together with the cooperation of health team members, will improve the quality of care and, consequently, the quality of life of these patients. Given the lack of a holistic palliative care program for CRC patients as well as the focus of some studies on the physical aspects of palliative care in these patients, this study is an attempt to find palliative care needs in CRC patients and develop a care program for them. As such, this study will be a step towards the promotion of a new palliative care in Iran.

## Conclusion

Designing a palliative care program for patients with CRC, in addition to controlling physical symptoms, other aspects of quality of life such as psychological, social, spiritual dimension will be considered and will improve the quality of life and health of patients in all dimensions.

## Data Availability

Not applicable in this study protocol. After finishing the enrollments of the participants, the data are available from the corresponding author on reasonable request.

## Abbreviations

CRC: Colorectal Cancer
QUAL: Qualitative study
Quan: Quantitative study
IUMS: Isfahan University of Medical Sciences
MACSA: ALA Center Control and Prevention Cancer
HCP: Health care providers
QLQ-CR29: Quality Of Life Questionnaire – Colorectal 29
EORTC: European Organization for Research and Treatment of Cancer
